# Declining comorbidity-adjusted mortality rates in English patients receiving maintenance renal replacement therapy

**DOI:** 10.1016/j.kint.2017.11.020

**Published:** 2018-05

**Authors:** Benjamin C. Storey, Natalie Staplin, Charlie H. Harper, Richard Haynes, Christopher G. Winearls, Raph Goldacre, Jonathan R. Emberson, Michael J. Goldacre, Colin Baigent, Martin J. Landray, William G. Herrington

**Affiliations:** 1 Medical Research Council Population Health Research Unit, Nuffield Department of Population Health (NDPH), University of Oxford, Oxford, UK; 2 Clinical Trial Service Unit and Epidemiological Studies Unit (CTSU), NDPH, University of Oxford, Oxford, UK; 3 Unit of Healthcare Epidemiology, Big Data Institute, Li Ka Shing Centre for Heath Information and Discovery, NDPH, University of Oxford, Oxford, UK; 4 Oxford Kidney Unit, Oxford University Hospitals NHS Foundation Trust, Oxford, UK

**Keywords:** epidemiological, mortality trends, RRT

## Abstract

We aimed to compare long-term mortality trends in end-stage renal disease versus general population controls after accounting for differences in age, sex and comorbidity. Cohorts of 45,000 patients starting maintenance renal replacement therapy (RRT) and 5.3 million hospital controls were identified from two large electronic hospital inpatient data sets: the Oxford Record Linkage Study (1965-1999) and all-England Hospital Episode Statistics (2000-2011). All-cause and cause-specific three-year mortality rates for both populations were calculated using Poisson regression and standardized to the age, sex, and comorbidity structure of an average 1970-2008 RRT population. The median age at initiation of RRT in 1970-1990 was 49 years, increasing to 61 years by 2006-2008. Over that period, there were increases in the prevalence of vascular disease (from 10.0 to 25.2%) and diabetes (from 6.7 to 33.9%). After accounting for age, sex and comorbidity differences, standardized three-year all-cause mortality rates in treated patients with end-stage renal disease between 1970 and 2011 fell by about one-half (relative decline 51%, 95% confidence interval 41-60%) steeper than the one-third decline (34%, 31-36%) observed in the general population. Declines in three-year mortality rates were evident among those who received a kidney transplant and those who remained on dialysis, and among those with and without diabetes. These data suggest that the full extent of mortality rate declines among RRT patients since 1970 is only apparent when changes in comorbidity over time are taken into account, and that mortality rates in RRT patients appear to have declined faster than in the general population.

Maintenance dialysis programs for end-stage renal disease (ESRD) began in the United Kingdom in the 1960s.^1^, ^2^, ^3^ Until the 1980s, renal replacement therapy (RRT; i.e., dialysis or kidney transplantation) was restricted to ESRD patients who were considered the most economically active, and those with diabetes or other comorbidities were often not referred or treated.^4^ This contrasts with the situation 50 years later, when the median age of patients starting maintenance RRT is 65 years and diabetes is the leading cause of ESRD.^5^

Examining long-term temporal mortality trends helps describe past and current serious health risks. Interpreting these trends is difficult in RRT populations because comparisons between patients treated for ESRD and other populations need to take account of the substantial secular changes in the prevalence of comorbid illnesses that influence both mortality^6^, ^7^, ^8^ and the likelihood of receiving RRT. To date, no large study has standardized mortality rates in treated ESRD and general population cohorts to the same comorbidity as well as age and/or sex structure. Therefore, although data from ESRD registries in the United States from 1977 to 2007,^9^ Europe from 1998 to 2007,^10^ Australasia from 1992 to 2005,^11^ and the UK from 2002 to 2011^5^ have all shown modest improvements in mortality for people with treated ESRD, it is unclear whether the magnitude of this change is comparable to that observed in the general population during the same period.^12^

The Oxford Record Linkage Study (ORLS) was established in 1963 and recorded information about all hospital inpatient admissions in Oxfordshire and surrounding counties covering about 5% of England (referred to as “Oxfordshire”).^13^ Hospital Episode Statistics (HES) succeeded ORLS and established nationwide coverage from 1998. Both data sets have been linked to national mortality registers, so we aimed to study mortality trends among new maintenance RRT patients and controls from the general population between 1970 and 2008 using novel approaches to ensure all cohorts could correct for changes in comorbidity over time. We also consider the effects of temporal changes in the availability of transplantation on mortality trends.

## Results

Between 1970 and 2008, 44,922 new ESRD patients started maintenance RRT (2192 in ORLS 1970–1996 and 42,730 from all-England HES 2000–2008), and 5,360,712 general population controls (532,019 from ORLS and 4,828,693 from HES) were identified. Indirect validation included observing closely matched numbers of kidney transplant operations recorded in HES and the UK Transplant Registry^14^ (Supplementary Table S1); closely matched cohort sizes, demographics and renal characteristics when HES data were compared with summary English data from the UK Renal Registry (Supplementary Table S2)^15^, ^16^, ^17^, ^18^; and similar age- and sex-adjusted 3-year mortality rates for ORLS/“HES Oxford” and for Oxford Kidney Unit (Supplementary Figure S1).

In Oxfordshire, the median age at start of maintenance RRT increased from 49 years (interquartile cut-offs 36–60 years) in 1970 to 1990 to 61 years (46–72 years) by 2006 to 2008. Consequently, while only one-quarter of patients starting RRT from 1970 to 1990 were aged ≥60 years, by 2006 to 2008 this proportion was more than one-half (Table 1). Of those starting RRT, the proportion who were female remained at about 40% across all time periods (Supplementary Figure S2A), but the proportion with any major comorbidity rose steeply from 1970 to 2008. In particular, diabetes prevalence among those starting RRT increased from 6.7% during 1970 to 1990 to 33.9% in 2006 to 2008, while prior vascular disease increased from 10.0% to 25.2% (Supplementary Figure S2B), constituting increases in peripheral arterial disease from 3.0% to 12.9%, major coronary disease from 2.6% to 8.3%, and admission for heart failure from 5.2% to 10.5% (Table 1). Prior cancer was recorded in 2.9% of RRT patients during 1970 to 1990 and 7.6% of patients during 2006 to 2008. The demographics and comorbidity of treated ESRD patients in Oxfordshire who started RRT between 2000 and 2008 were broadly similar to those observed in the rest of England (Table 1).

**Table 1:** Baseline characteristics of newly treated end-stage renal disease patients, by year

Characteristics	Year groups							
	Oxfordshire					All-England		
	Oxford Record Linkage Study		Hospital Episode Statistics (Oxford)			Hospital Episode Statistics (All-England)		
	1970–1990	1991–1996	2000–2002	2003–2005	2006–2008	2000–2002	2003–2005	2006–2008
*N*	1220	972	700	750	878	13,178	13,606	15,946
**Demographics**								
Female	40.2%	38.0%	41.1%	35.9%	37.7%	39.5%	37.9%	38.5%
Median age (yr)	49 (36–60)	59 (44–69)	61 (45–72)	61 (45–72)	61 (46–72)	61 (47–71)	62 (47–72)	63 (49–73)
18–40	30.2%	18.7%	18.7%	18.7%	15.9%	15.8%	14.8%	13.0%
40–50	21.2%	15.6%	13.6%	13.1%	14.7%	13.5%	13.4%	12.9%
50–60	23.5%	17.2%	15.4%	16.4%	17.1%	17.8%	16.6%	17.2%
60–70	16.8%	24.2%	20.7%	20.8%	22.1%	23.1%	22.9%	22.7%
70–80	7.9%	20.5%	24.4%	23.5%	19.8%	23.6%	24.1%	24.5%
≥80	0.3%	3.8%	7.1%	7.6%	10.4%	6.1%	8.2%	9.7%
Ethnicity^a^, ^c^								
White	-	-	86.8%	86.4%	84.0%	82.1%	81.2%	79.9%
Black	-	-	3.5%	3.5%	4.7%	6.3%	6.5%	6.9%
South Asian	-	-	7.4%	6.3%	7.2%	8.2%	8.3%	8.7%
Other	-	-	2.3%	3.8%	4.0%	3.4%	3.9%	4.4%
Unknown	-	-	131	36	35	1,694	962	745
**Comorbidities**								
Diabetes	6.7%	16.8%	24.4%	29.2%	33.9%	25.7%	29.9%	34.3%
Vascular	10.0%	18.3%	22.3%	24.7%	25.2%	25.2%	26.5%	28.3%
Major coronary disease	2.6%	4.2%	5.1%	7.2%	8.3%	6.1%	7.0%	7.7%
Congestive heart failure	5.2%	8.5%	9.9%	10.8%	10.5%	11.7%	12.3%	12.8%
Cerebrovascular disease	1.4%	2.2%	3.1%	2.8%	3.5%	3.3%	3.4%	3.4%
Peripheral arterial disease	3.0%	7.8%	11.3%	11.5%	12.9%	12.0%	12.5%	14.2%
Nonvascular^b^	7.8%	14.4%	18.3%	21.7%	24.9%	21.7%	25.0%	27.5%
Liver disease	0.5%	0.4%	1.7%	1.1%	2.3%	1.6%	2.0%	2.8%
Cancer	2.9%	4.6%	5.3%	8.9%	7.6%	6.4%	7.8%	8.3%
Chronic obstructive pulmonary disease	1.3%	2.9%	6.3%	6.5%	10.3%	8.3%	10.0%	12.1%
Peptic ulcer disease	1.6%	2.3%	2.7%	1.9%	1.9%	2.3%	2.3%	2.0%
Connective tissue disease	2.0%	4.3%	3.1%	4.4%	4.9%	4.7%	5.0%	4.8%
**Renal characteristics**^c^								
Initial renal replacement therapy modality								
Dialysis	94.6%	92.6%	93.7%	92.7%	91.6%	94.5%	94.3%	93.6%
Transplant	5.4%	7.4%	6.3%	7.3%	8.4%	5.5%	5.7%	6.4%
Primary renal diagnosis (presumed)								
Diabetic kidney disease	1.6%	8.4%	20.0%	22.5%	22.1%	19.1%	20.1%	20.4%
Glomerulonephritis	9.3%	14.1%	9.3%	10.8%	14.5%	10.8%	12.2%	14.1%
Polycystic kidney disease	10.5%	8.4%	8.6%	7.5%	10.4%	9.2%	8.6%	8.9%
Other known diagnosis/unknown	78.5%	69.0%	62.1%	59.2%	53.1%	60.9%	59.1%	56.6%

Excludes patients dying within 90 days. Data are *n* or % or median (interquartile range).

a Ethnicity only recorded in Hospital Episode Statistics (92% complete) with percentages quoted only for those with a known ethnicity.

b Also includes hemiplegia or paraplegia.

c Not used for standardization. Baseline characteristics of general population hospital controls are in Supplementary Table S3.

Compared with new ESRD patients, general population controls were on average younger and more likely to be female. General population controls in the later time periods were older and had more comorbidity than general population controls from the earlier periods (Supplementary Table S3).

### All-cause mortality

**Figure 1:**
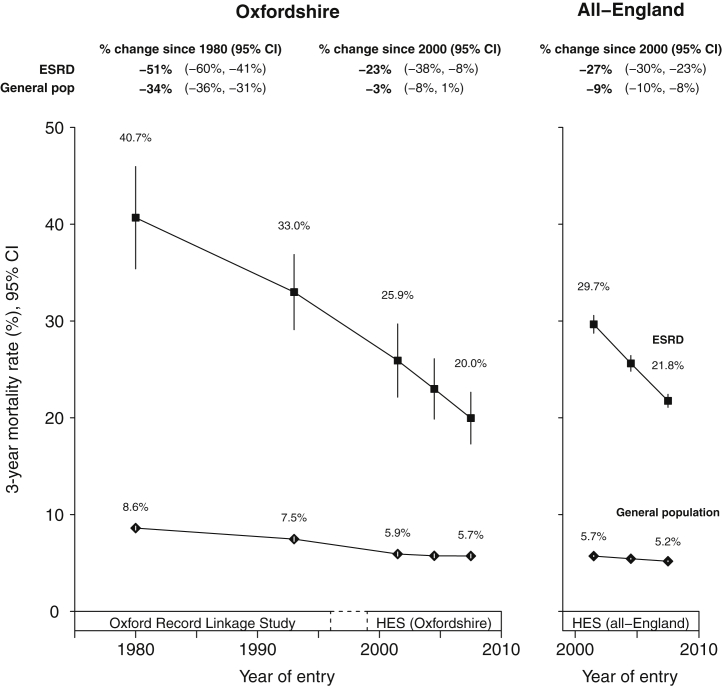
**Standardized 3-year mortality rates in newly treated end-stage renal disease (ESRD) patients and general population hospital controls.** Standardized to the age, sex, and comorbidity structure of an “average” 1970 to 2008 renal replacement therapy population (see Supplementary Table S6 for characteristics). Excludes patients dying within 90 days. Year of entry is year of starting renal replacement therapy or year of relevant general population hospital controls admission. Rates plotted at midpoint of each year group. CI, confidence interval; HES, Hospital Episode Statistics.

Of the 1220 new ESRD patients starting RRT in 1970 to 1990, 267 (crude 3-year mortality rate 24.8%) died within the first 3 years. For the 878 Oxfordshire patients and 15,946 all-England patients starting RRT in 2006 to 2008, 221 (28.7%) and 4482 (38.2%) died within 3 years, respectively. Crude mortality rates—which do not take account of secular changes in age, sex, or comorbidity of those who received maintenance RRT—showed an average increase in mortality between 1970 and 1996, followed by the beginnings of a decline (Supplementary Figure S3A). After standardization by age and sex, however, a continuous decline in 3-year mortality rates from 1970 became evident (Supplementary Figure S3B), which steepened further when diabetes and other comorbidities were accounted for (Supplementary Figure S3C). On a relative scale, this corresponded to a 25-year 51% (95% confidence interval [CI], 41%–60%) decline in standardized 3-year mortality rates since about 1980 (Figure 1). Examination of 1-year, 2-year, 3-year, and 5-year mortality rates also showed steep declines in mortality rate with time (Supplementary Figure S4).

Standardized 3-year all-cause mortality rates in the general population were substantially lower than for new ESRD patients, and declined somewhat less steeply: from 8.6% during 1970 to 1990 (15,158 deaths in 406,897 people) to 5.7% (3885 deaths in 79,593 people) during 2006 to 2008. On a relative scale, this represented a 25-year reduction of 34% (95% CI 31%–36%; Figure 1). All-England data from 2000 mirrored findings in Oxfordshire data from the same period (Figure 1).

**Figure 2:**
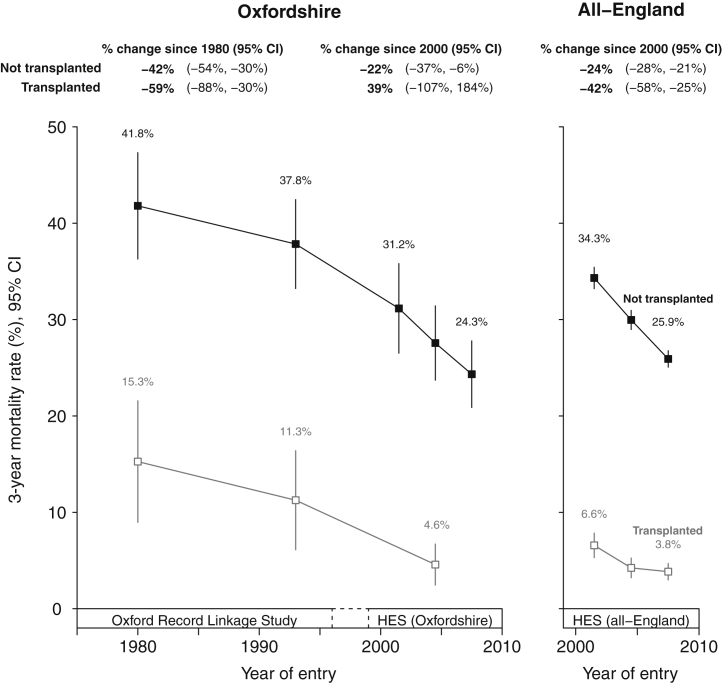
**Standardized 3-year mortality rates in newly treated end-stage renal disease patients, stratified by whether patient underwent transplant within 3 years of starting renal replacement therapy.** Standardized to the age, sex, and comorbidity structure of an “average” 1970 to 2008 renal replacement therapy population (see Supplementary Table S6 for characteristics). Excludes patients dying within 90 days. Year of entry is year of starting renal replacement therapy or year of relevant general population hospital controls admission. Rates plotted at midpoint of each year group. CI, confidence interval; HES, Hospital Episode Statistics.

Kidney transplantation was introduced in Oxfordshire in 1975. The 3-year standardized mortality rate among these early transplant recipients was substantially lower than for those who remained on dialysis (15.3% vs. 41.8% during 1970–1990), and fell over time such that the 2000 to 2008 3-year standardized mortality rates for transplanted patients were 4.6% (Figure 2). Despite increased availability of transplantation over time (the proportion of patients receiving a transplant within 3 years of needing to start RRT increased from 26% to 30% between 1970–1990 and 2006–2008), 3-year mortality also substantially and continually declined among ESRD patients who remained on dialysis. The rates of improvements in mortality were similar in both the Oxfordshire and all-England data.

### All-cause mortality by diabetes

**Figure 3:**
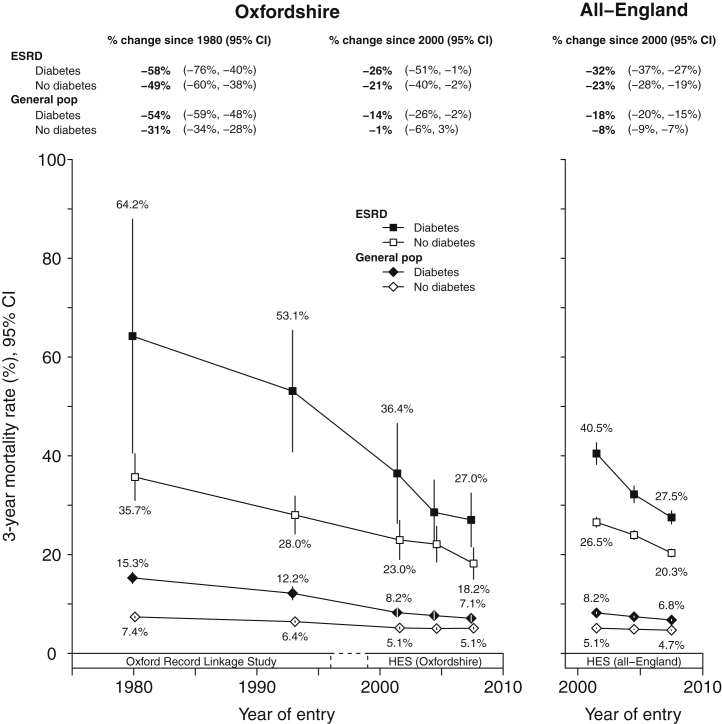
**Standardized 3-year mortality rates in newly treated end-stage renal disease (ESRD) patients and general population hospital controls, stratified by prior diabetes.** Standardized to the age, sex, and comorbidity structure of an “average” 1970 to 2008 RRT population (see Supplementary Table S6 for characteristics). Excludes patients dying within 90 days. Year of entry is year of starting renal replacement therapy or year of relevant general population hospital controls admission. Rates plotted at midpoint of each year group. CI, confidence interval; HES, Hospital Episode Statistics.

In the general population, there were steeper reductions in mortality over time in people with diabetes (heterogeneity *P* < 0.0001 for Oxfordshire and *P* < 0.0001 for all-England). The same was not observed among treated ESRD patients in Oxfordshire over 25 years since 1980 (heterogeneity *P* = 0.41), but there was evidence of steeper declines in mortality rates among people with diabetes from 2000 in England (heterogeneity *P* = 0.01). The absolute difference in mortality rates between those with and without diabetes has therefore become substantially smaller between 1970 and 2011 (Figure 3).

### Vascular mortality

**Figure 4:**
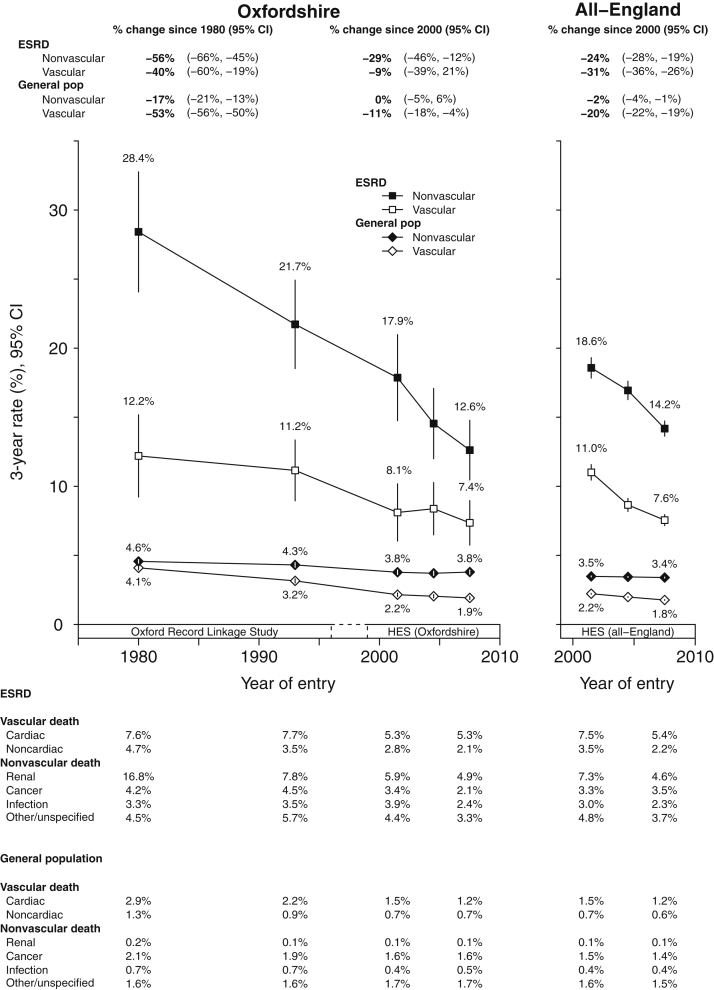
**Standardized 3-year vascular and nonvascular mortality rates in newly treated end-stage renal disease (ESRD) patients and general population hospital controls.** Standardized to the age, sex, and comorbidity structure of an “average” 1970 to 2008 RRT population (see Supplementary Table S6 for characteristics). Excludes patients dying within 90 days. Year of entry is year of starting renal replacement therapy or year of relevant general population hospital controls admission. Rates plotted at midpoint of each year group. CI, confidence interval; HES, Hospital Episode Statistics.

Among new ESRD patients, 3-year mortality rates from vascular causes fell from 12.2% between 1970 to 1990 to 7.4% by 2006 to 2008, representing a 25-year relative reduction of about 40% (95% CI 19%–60%) since 1980, which included about a 31% (95% CI 2%–60%) reduction in cardiac mortality and 55% (95% CI 28%–82%) reduction in noncardiac vascular mortality (Figure 4).

In general population controls, 3-year mortality from vascular mortality declined from 4.1% in the 1970 to 1990 group to 1.9% by 2006 to 2008. This represented a relative 25-year decline in 3-year vascular mortality of 53% (95% CI 50%–56%), which included a 58% (95% CI 55%–61%) decline in cardiac and 45% (95% CI 40%–50%) decline in noncardiac vascular mortality (Figure 4). Between 1970 and 2011, declines in cardiac mortality have therefore been steeper in the general population than in new ESRD patients. Again, all-England data from 2000 to 2011 mirrored findings from Oxfordshire in 2000 to 2011.

### Nonvascular mortality

In new ESRD patients, 3-year mortality from nonvascular causes declined steeply and continuously since 1970 from 28.4% in the 1970 to 1990 group to 12.6% by 2006 to 2008 (Figure 4). On a relative scale this represented a 25-year decline of 56% (95% CI 45%–66%) since 1980. The commonest underlying nonvascular causes of death were from renal failure or its causes (e.g., deaths in which chronic, diabetic, hypertensive, and polycystic kidney diseases initiated the train of terminal events that led to death). Such mortality fell from 16.8% to 4.9% between the 1970 to 1990 and 2006 to 2008 groups, a relative decline of 71% (95% CI 61%–81%). Declines in other common nonvascular causes were more modest. These included a reduction of 27% in infectious mortality (95% CI –14% to 68%; absolute decline from 3.3% to 2.4%) and a reduction of 50% in cancer mortality (95% CI 21%–80%; absolute rates 4.2% and 2.1%; Figure 4).

In general population controls, the declines in 3-year nonvascular mortality were more modest than the corresponding declines in new ESRD patients. Three-year standardized mortality rates fell from 4.6% in 1970 to 1990 to 3.8% by 2006 to 2008, which on a relative scale represents a 25-year 17% (95% CI 13%–21%) decline since 1980. This included a 25-year 26% relative reduction in death from cancer (95% CI 20%–31%; absolute decline from 2.1% to 1.6%), and 30% relative reduction in infection-related mortality (95% CI 22%–37%; absolute decline from 0.7% to 0.5%; Figure 4).

In treated ESRD patients, the steeper proportional declines in nonvascular mortality compared with those in the general population (56% vs. 17%, Figure 4), and shallower declines in vascular mortality (40% vs. 53%, respectively) resulted in the proportion of all deaths ascribed to vascular disease rising from 29.9% in 1970 to 1990 to 36.8% in 2006 to 2008, while the proportion of all deaths ascribed to vascular disease in the general population fell from 47.5% to 33.3% over the same period.

## Discussion

We have used large cohorts derived from routine hospital admission data sets established before the start of maintenance RRT programs to compare changes in cause-specific mortality among people with newly treated ESRD and contemporaneous general population controls, taking account of the major changes in age and comorbid illnesses of those selected to start RRT since 1970. Three-year absolute mortality rates from many causes have remained high among people on maintenance RRT, but on a relative scale, overall mortality has halved. This decline is substantially steeper than the one-third decline observed in the general population. As those on RRT are at much higher mortality risk than the general population, this also translates into substantially larger reductions in absolute mortality rates.

An important finding from this study is that the reported reductions in mortality rates have declined faster than reported by ESRD registries in the United States from 1977 to 2007,^9^ Europe 1998 to 2007,^10^ Australasia 1992 to 2005,^11^ and the UK 2002 to 2011.^5^ These registry studies may have underestimated improvements in mortality by virtue of not being able to adjust for temporal changes in serious vascular and nonvascular comorbidities. Our comorbidity-adjusted estimates suggest relative mortality declines of perhaps 30% over the 10 years from the mid-1990s, which is larger than the approximately 20% declines evident from contemporaneous European registry data *without* such comorbidity adjustment.^19^, ^20^ Our results from HES data were, however, almost identical to the relative declines in comorbidity-adjusted mortality rates reported by a 2002 to 2006 study that used UK Renal Registry HES-linked data.^21^

Over the last 40 years, there has been a progressive and steep increase in the proportion of people with diabetes who start RRT treatment for ESRD. We found evidence that mortality rates have fallen faster among people with diabetes both in the general population and in those on maintenance RRT, meaning the absolute gap in mortality rates between those with and without diabetes has progressively closed over the last few decades.

This study includes data in the 25 years before RRT registries had complete nationwide coverage in England. Over the early period, the numbers of people undergoing RRT progressively increased, and short-to-medium mortality was still attributed in large part to renal failure or its causes. This renal mortality rate appears to have fallen by more than one-half over the last 40 years. If this is true, kidney transplantation may have been a key intervention in reducing such mortality.^22^ By 2000, 25 years after the first kidney transplant in Oxford,^23^ standardized 3-year mortality rates among those selected to receive a kidney transplant were as low as 4% to 5%. However, those remaining on dialysis have also experienced substantial improvements in mortality rates over time, which could be attributable to multiple incremental improvements in the way renal care has been delivered in dialysis units and/or improvements in the way patients are prepared for RRT.^24^, ^25^

In contrast to the early improvements in renal mortality, reductions in mortality rates from infections were more delayed, beginning from the late 1990s. More recent improved understanding of immunosuppression and focus on infection control measures including hand hygiene protocols, flushed connection systems for peritoneal dialysis catheters,^26^ emphasis on natural arteriovenous hemodialysis access,^27^, ^28^ the introduction of antibiotic hemodialysis catheter locks,^29^, ^30^ and proactive vaccination programs^31^ may all have contributed.

Our finding that mortality from vascular disease has declined less steeply among treated ESRD populations than general populations corroborates similar observations made in Australasia between 1992 and 2005.^11^ These UK results now demonstrate that this lesser decline in vascular mortality appears to result from slow declines in cardiac mortality. The reasons why improvements in cardiac mortality rates in treated ESRD populations have been slower than the rapid declines observed in general populations (both in this study and in other national representative data^32^) cannot be tested in the present study. Other studies have found that effective interventions to reduce vascular mortality in people at high risk^33^, ^34^, ^35^ may be less effective in ESRD populations (e.g., lowering low-density lipoprotein cholesterol^36^), and interventions for renal-specific risk factors (e.g., renal anemia,^37^ low dialysis dose,^38^ and hyperparathyroidism^39^) do not have clear cardiovascular benefits. Studies also suggest that there has been underuse of coronary intervention in people with chronic kidney disease.^40^ Identification of the causes of high vascular mortality rates in ESRD patients should remain a research priority.

Using indirect methods of validation with data from registries of UK RRT activity and the Oxford Kidney Unit, we have shown that routinely collected hospital admission data, although not completely free from error (Supplementary Tables S1 and S2; Supplementary Figure S1), can provide representative and reliable descriptions of changes in mortality rates over many decades, with our results mirroring recent HES-linked UK Renal Registry 2002 to 2006 findings.^21^ A limitation of these data, however, was the inability to *directly* validate the cohort. Another limitation of these data is that the general population controls were selected for having been hospitalized for minor conditions. This was necessary because it enabled adjustment for comorbidity and thus reliable comparisons between the different populations. We cannot guarantee, therefore, that the mortality rates in hospital controls were completely representative of mortality rate declines in unselected Oxfordshire and English populations. Another limitation was the lack of information on certain exposures that may have changed substantially over time and influenced mortality, such as cigarette smoking. Finally, completion of death certificates may have varied with time, and in particular, some deaths due to vascular causes may have been attributed to renal disease, infection, or other nonvascular causes (and vice versa) and nonatherosclerotic causes of cardiac death may have gone unrecognized in the 1970s to 1990s. Moreover, we were unable to ascertain which renal deaths were from withdrawal of dialysis and which were the results of direct complications. Nevertheless, a key strength of this study is that cause-specific mortality data from all the cohorts share the same certification and coding principles in any given year, making comparisons between ESRD and general populations more reliable.^41^, ^42^, ^43^

In conclusion, the full extent of mortality rate declines among RRT patients since 1970 is only apparent when changes in comorbidity over time are taken into account. This approach suggests mortality rates in RRT patients have halved since 1970, faster than declines in mortality in the general population. Declines in 3-year mortality rates were evident among those who received a kidney transplant and those who remained on dialysis. However, among those undergoing RRT with or without diabetes, high residual mortality risk from both vascular and nonvascular causes remains.

## Methods

The Central and South Bristol Multi-Centre Research Ethics Committee (04/Q2006/176) granted ethical approval for these analyses of linked hospital inpatient data. Retrospective cohorts of new maintenance RRT patients and general population hospital controls were derived from 2 routinely collected hospital inpatient data sets with linkage to national mortality data. The ORLS collected information on hospital admissions in Oxfordshire from 1963, expanding to surrounding counties to cover a population of 2.5 million.^44^ Nationwide individual patient-linked HES data replaced ORLS in 1998, recording information about inpatient admissions from all National Health Service hospitals in England. Analyses include a period from January 1, 1965, to December 31, 2011 (cohort follow-up started from January 1, 1970, with data prior to this being used to determine baseline comorbidities).

Both ORLS and HES record detailed information about hospital admissions including patient demographics, dates of admission and discharge, admitting specialty, primary diagnosis and relevant secondary diagnoses (all coded using the International Statistical Classification of Diseases and Related Health Problems [ICD] versions 7 to 10), and all inpatient procedures accompanied by their dates (coded using the Office of Population Censuses and Surveys [OPCS] Classification of Surgical Operations and Procedures versions 2 to 4).

We developed algorithms incorporating diagnostic, procedural, and specialty codes relevant to renal disease, dialysis, and transplantation to identify adults aged ≥18 years in ORLS who started RRT between 1970 and 1996, and in HES between 2000 and 2008. Those patients whose records indicated dialysis was for acute kidney injury or who died within 90 days of starting RRT were excluded (as is standard in the study of incident ESRD cohorts). For full details of cohort derivations, see Supplementary Figures S5 and S6. To allow mortality rates from the newly treated ESRD cohort to be compared with a group of contemporaneous adults, hospital controls who were never recorded as undergoing RRT were selected so as to be reasonably representative of the general population by using admissions for a range of minor conditions including inguinal hernias, soft-tissue knee complaints, tonsillectomy, etc. (full list of conditions in Supplementary Material). Hospital controls provided the advantage that comorbidity could be identified from admission records (information that is incompletely recorded in vital statistics). Baseline information on age, sex, and ethnicity (categorized into White, Black, South Asian, other, and unknown, and only reported in HES) was extracted.^45^ A presumed primary renal diagnosis (polycystic kidney disease, glomerulonephritis, diabetic kidney disease, or other and/or unknown cause), initial RRT modality (dialysis or transplant) and comorbidities based on the Charlson index^21^, ^46^ were identified from diagnostic and procedural codes on admission records at the time of entry into the cohort and for a fixed period of retrospective follow-up beforehand. For the purpose of adjustment, comorbid illnesses were classified as (i) diabetes mellitus (combining types 1 and 2); (ii) vascular disease, including major coronary disease, heart failure, cerebrovascular disease, and peripheral arterial disease; and (iii) serious nonvascular disease including liver disease, cancer, chronic obstructive pulmonary disease (COPD), peptic ulcer disease, hemi- or paraplegia, and connective tissue disease (definitions in Supplementary Table S5).

We assessed the reliability of routine hospital admission data for the identification of newly treated ESRD by comparing the number of transplants in ORLS and HES with the UK Transplant Registry (Supplementary Table S1)^14^, the cohort sizes and characteristics with UK Renal Registry (Supplementary Table S2) annual reports,^15^, ^16^, ^17^, ^18^ and data (including mortality rates) collected from Oxford Kidney Unit databases compiled prospectively since 1967 (Supplementary Figure S1).

Subsequent mortality was identified from linked national mortality data. The primary outcome was all-cause mortality, and secondary outcomes were cause-specific mortality identified from the underlying causes of death and separated into vascular (cardiac and noncardiac) and nonvascular mortality (renal disease [i.e., death from renal failure or its causes], cancer, infection, and other and/or unspecified; definitions in Supplementary Table S4).

### Statistical analyses

Patient follow-up was separated by year of cohort entry into 5 groups: 1970 to 1990, 1991 to 1996, 2000 to 2002, 2003 to 2005, and 2006 to 2008 (i.e., there was a gap between the 2 cohorts between 1997 and 1999 where there was transition into All-England HES). The different number of years covered by each group ensured similar numbers of patients in the 2 ORLS groups (1970–1996) and, separately, in the 3 HES groups (2000–2008). All-cause and cause-specific mortality rates for each group were estimated using Poisson regression adjusted for age, sex, and comorbidities. Three-year mortality rates are presented because it ensured that data from those starting dialysis as late as 2008 could be included. Age was included as a continuous variable using linear and quadratic terms. To account for the Poisson regression assumption that the mean and variance of the rates are equal, robust standard errors were calculated.^47^ Marginal standardization^48^ was used to adjust mortality rates to the characteristics of an “average” 1970 to 2008 RRT population, defined using the entire ORLS RRT cohort and a random sample from each of the HES year groups such that the standard population had approximately equal numbers of RRT patients from each decade (characteristics in Supplementary Table S6; see Additional Statistical Methods for further details of methods). To allow for comparisons of change in mortality over time between the ESRD and general population cohorts, percentage change in 3-year mortality rates between the 1970 to 1990 and 2006 to 2008 groups (i.e., over approximately 25 years) were presented with 95% CI for the ORLS and a HES cohort that closely matched the ORLS catchment area (defined using Area of Residence District Health Authority Codes for Oxfordshire, Berkshire, Buckinghamshire, and Northamptonshire, and referred to as “HES Oxford” in figures). For the all-England HES and the HES Oxford cohorts, percentage changes in 3-year mortality rates between the 2000 to 2002 and 2006 to 2008 groups (i.e., over about 10 years) are presented so mortality trends from Oxfordshire and surrounding counties can be compared with all-England data.

To explore mortality rates among those who received a transplant and those who did not, subsequent analyses were stratified by including an interaction term between year group and transplantation status by 3 years. This allowed estimation of separate rates for transplant recipients and those who remained on dialysis. Subgroup analyses by prior diabetes and other baseline characteristics were performed using a similar method, and are accompanied by standard heterogeneity tests that compare relative reductions in mortality over time between the subgroups.

In sensitivity analyses, 1-year, 2-year, 4-year, and 5-year mortality rates were also calculated for comparison (Supplementary Figure S4). Three-year mortality rates standardized to a 2006 to 2008 English RRT population were also provided. A sensitivity analysis using an exposure-matched cohort (matching on age [nearest year], sex, prior diabetes mellitus, prior vascular disease, prior nonvascular disease, and year group) was also performed (see Additional Statistical Methods and Supplementary Figure S7). All analyses used SAS version 9.3 (SAS Institute; Cary, NY) and R software version 3.2.1.

## Disclosure

The funders had no role in study design, data collection, data analysis, data interpretation, or writing of the report.

The Clinical Trial Service Unit has a staff policy of not accepting honoraria or other payments from the pharmaceutical industry, expect for the reimbursement of costs to participate in scientific meetings (www.ctsu.ox.ac.uk). RH reported grants from Merck, Novartis, and Pfizer, outside the submitted work. CGW reported grants from Kidney Research UK and Roche, outside the submitted work. CB and MJL reported grants from British Heart Foundation, Medical Research Council, Cancer Research UK, Merck, Novartis, and Pfizer during the conduct of the study. All the other authors declared no competing interests.

## Supplementary Material

Additional Statistical Methods

Table S1 Number of kidney transplant operations in England recorded in all-England Hospital Episode Statistics and the UK Transplant Registry, by month and year.

Table S2 Baseline characteristics of all-England adults being treated for end-stage renal disease, recorded in all-England Hospital Episode Statistics and the UK Renal Registry by year.

Table S3 Baseline characteristics of general population hospital controls, by year.

Table S4 Coding of death categories by International Classification of Disease (ICD) revision.

Table S5 Coding of comorbidity by International Classification of Disease (ICD) diagnoses and Office of Population Censuses and Surveys (OPCS) procedures.

Table S6 Baseline characteristics of end-stage renal disease populations used for standardization.

Table S7 Diagnostic and procedural codes used to identify general population controls.

Figure S1 Age- and sex-standardized 3-year mortality rates in newly treated end-stage renal disease patients and general population hospital controls in Oxfordshire.

Figure S2 Baseline characteristics of newly treated end-stage renal disease patients, by year.

Figure S3 Crude and standardized 3-year mortality rates in newly treated end-stage renal disease patients and general population hospital controls, by year and reference population.

Figure S4 Standardized 1- to 5-year survival probabilities in newly treated end-stage renal disease patients.

Figure S5 Treated end-stage renal disease cohort derivation (all-England HES 2000–2008).

Figure S6 Treated end-stage renal disease cohort derivation (Oxford Record Linkage Study 1970–1996).

Figure S7 Standardized 3-year mortality rates in newly treated end-stage renal disease patients and general population hospital controls, by year and matching method.
