# Supplementary material for: Declining comorbidity-adjusted mortality rates in English patients receiving maintenance renal replacement therapy
Source: Kidney Int. 2018 May;93(5):1165–74. doi: 10.1016/j.kint.2017.11.020 (PMC5912929; doi:10.1016/j.kint.2017.11.020)
Supplement: Additional Statistical Methods [file mmc1.pdf]

Supplementary Appendix: Additional statistical methods

Poisson regression was used to adjust for differences in age, sex and comorbidity structures between the year groups and marginal standardization was used to estimate mortality rates for particular populations (see below). In each year group, this approach gives estimated mortality rates for hospital controls and RRT patients standardized to the same age, sex and comorbidity structure as the 'average' 1970-2008 RRT population.

Year groups used in analyses

Patient follow-up was separated by year of cohort entry into five groups across the two cohorts:

Oxford Record Linkage Study (ORLS):

- 1970-1990
- 1991-1996

All-England Hospital Episode Statistics (HES):

- 2000-2002
- 2003-2005
- 2006-2008

There was a gap between the two cohorts from 1997 to 1999 where there was transition into All-England HES. The different number of years covered by each group ensured similar numbers of patients in each of the two ORLS groups and, separately, in each of the three HES groups.

Marginal standardization and definition of the 'average' 1970-2008 RRT population

Separately for patients starting RRT and the general population hospital controls, mortality rates were modelled using Poisson regression adjusted for grouped year of cohort entry (in the 5 categories defined above), age (linear and quadratic terms), sex and comorbidities (diabetes, vascular disease and serious non-vascular disease). Robust standard errors were calculated[1] which have been shown to be an appropriate method of dealing with the violation of the Poisson regression assumption of equidispersion.[2] The estimated mortality rates obtained from Poisson regression need to be applicable to the population being studied. The commonly used approach of predicted rates based on mean values of all covariates does not correspond to any real-life observations and these rates may be non-sensical in the presence of binary covariates.[3] Marginal standardization gives predicted rates summed to a weighted average that reflects the confounder distribution in the 'target population'.[3]

Definition of the 'average' 1970-2008 RRT population

The target population chosen for these analyses was an 'average' 1970-2008 RRT population, defined using the entire ORLS RRT cohort and a random sample from each of the full all-England HES year groups, such that the standard population had approximately equal numbers of RRT patients from each decade (baseline characteristics in Supplemental Table 6).

Estimating standardized mortality rates (1970-2008) for the RRT population

The following describes the marginal standardization procedure for the first year group in our analyses, ORLS 1970-1990. The standardized mortality rate obtained is the average mortality rate that we would have expected to observe between 1970 and 1990 <u>if the patients that started RRT in this period had the same age, sex and comorbidity structure as the 'average' 1970-2008 RRT population (see above).</u>

- **Step 1:** Assume that every individual in the target population (i.e. the 'average' 1970-2008 RRT population) entered the cohort in 1970-1990. The binary covariate representing this year group is therefore set to 1 and

the covariates for the other 4 groups (ie, ORLS 1991-96, HES 2000-02, HES 2003-05, HES 2006-08) are all set to zero.

- **Step 2:** All other covariates used in the model (i.e. age, sex, and comorbidities) are kept at their observed values.
- **Step 3:** The regression coefficients estimated from the Poisson regression model for RRT patients are then applied to each of the individuals to give the predicted probability of the outcome (i.e. death) for each patient.
- **Step 4:** These are summed to get a total expected death 'count' between 1970 and 1990.
- **Step 5:** This total was then divided by total units of follow-up (i.e. one unit of follow-up was 3 years in the primary analyses) and multiplied by 100 to give the average 3-year % mortality over the period 1970 – 1990.
- **Step 6:** The standard error for this rate was generated using the delta method.[4]

This procedure was then repeated for ORLS 1991-1996, HES 2000-02, HES 2003-05 and HES 2006-08 (except now with the appropriate alternative year group set to 1 in step 1). Analyses were done by SAS version 9.3, using previously published macros.[4]

Estimating standardized mortality rates (1970-2008) for general population hospital controls

The procedure used to standardize the rates for the general population hospital controls is similar to that outlined above for the RRT population. The 'average' 1970-2008 RRT population is still used as the target population, so that we estimate the average mortality rate that we would have expected to observe for the general population hospital controls in a given year group if they had the same age, sex and comorbidity structure as the 'average' 1970-2008 RRT population. This allows direct comparison with the standardized mortality rates for the RRT population. The only difference in the approach is that the regression coefficients used in Step 3 come from the Poisson regression model for the general population hospital controls instead.

Sensitivity analysis: Using 'matched controls' rather than all controls

A sensitivity analysis matching hospital controls to RRT patients based on age (nearest year), sex and comorbidities (prior diabetes, prior vascular disease and other prior non-vascular disease) and year group was also conducted (1:1 matching). The small number of RRT patients (n=125) for whom a matching hospital control could not be found were exclud from this analysis.[a]

References for additional statistical methods

1. Diggle PJ, Heagerty P, Liang K-Y, Zeger SL. Robust estimation of standard errors. *Analysis of Longitudinal Data*. Oxford: OUP; 2002:70 - 80.
2. Cameron AC, Trivedi PK. Microeconometrics Using Stata. College Station, TX: Stata Press; 2009.
3. Muller CJ, MacLehose RF. Estimating predicted probabilities from logistic regression: different methods correspond to different target populations. *International Journal of Epidemiology.* Jun 2014;43(3):962-970.
4. Zou GY. Assessment of risks by predicting counterfactuals. *Statistics in Medicine* 2009;28:3761–81.

[a] Note that in this analysis (shown in supplemental figure 7) the 95% confidence intervals around the 3-year mortality rates for the hospital controls are narrower than for the RRT cohort (despite there being fewer deaths among the hospital controls). The variance-covariance matrix from the Poisson regression model for the general population hospital controls contained larger variances than that for the RRT cohort, as we would expect. However, these variance-covariance matrices are pre- and post-multiplied by the Jacobian matrix of first order partial derivatives of the estimator when applying the delta method. As the mortality rate is substantially lower in the hospital controls, the elements of the Jacobian matrix are also much smaller, leading to smaller standard errors for the mortality rates in this population.
