# Supplementary material for: Declining comorbidity-adjusted mortality rates in English patients receiving maintenance renal replacement therapy
Source: Kidney Int. 2018 May;93(5):1165–74. doi: 10.1016/j.kint.2017.11.020 (PMC5912929; doi:10.1016/j.kint.2017.11.020)
Supplement: Table S1 — Number of kidney transplant operations in England recorded in all-England Hospital Episode Statistics and the UK Transplant Registry, by month and year. [file mmc2.pdf]

Supplemental table 1: Number of kidney transplant operations in England recorded in all-England Hospital Episode Statistics and the UK Transplant Registry†, by month and year

	2000		2001		2002		2003		2004		2005		2006		2007		2008		2009		2010		2011		2000-2011	
**Month**	**HES**	**UKTR**	**HES**	**UKTR**	**HES**	**UKTR**	**HES**	**UKTR**	**HES**	**UKTR**	**HES**	**UKTR**	**HES**	**UKTR**	**HES**	**UKTR**	**HES**	**UKTR**	**HES**	**UKTR**	**HES**	**UKTR**	**HES**	**UKTR**	**HES**	**UKTR**
Jan	99	104	100	119	120	129	140	145	130	147	100	104	109	108	145	136	164	157	178	169	213	208	191	185		
Feb	93	102	89	95	70	81	99	103	144	155	97	101	136	145	133	131	145	145	161	153	176	166	182	178		
Mar	134	154	120	128	87	99	104	113	130	145	114	118	118	132	143	146	152	150	173	170	183	197	170	174		
Apr	99	106	88	96	94	97	97	100	119	127	141	140	126	123	129	126	165	166	166	157	175	171	161	154		
May	102	109	130	144	107	107	105	117	126	129	139	143	152	150	159	153	166	161	180	178	167	171	191	185		
Jun	113	124	102	105	112	124	111	125	109	107	138	138	143	149	149	143	144	142	178	171	191	186	171	166		
Jul	99	104	120	136	110	113	105	112	113	121	141	143	140	137	158	156	183	186	186	182	181	172	203	204		
Aug	104	114	112	124	104	112	98	113	115	108	115	111	136	133	145	137	149	148	169	168	162	155	175	170		
Sep	81	88	98	104	124	129	151	147	111	122	126	131	159	157	131	133	176	178	169	167	162	166	194	197		
Oct	100	120	125	137	149	156	111	121	145	145	132	127	142	138	154	144	211	208	210	206	193	193	194	187		
Nov	121	129	107	117	152	158	119	127	114	119	109	114	161	156	165	167	171	176	194	196	221	217	199	195		
Dec	113	127	98	108	113	121	98	109	141	141	138	139	158	148	157	151	168	166	187	178	186	186	200	192		
**Total**	1258	1381	1289	1413	1342	1426	1338	1432	1497	1566	1490	1509	1680	1676	1768	1723	1994	1983	2151	2095	2210	2188	2231	2187	**20248**	**20579**
**Absolute**	-123		-124		-84		-94		-69		-19		4		45		11		56		22		44		**-331**	
**%**	-8.9%		-8.8%		-5.9%		-6.6%		-4.4%		-1.3%		0.2%		2.6%		0.6%		2.7%		1.0%		2.0%		**-1.6%**	

HES = Hospital Episode Statistics. UKTR = UK Transplant Registry. Multi-visceral organ transplants excluded. †Incorporated into National Health Service Blood and Transplant (NHSBT) [http://www.nhsbt.nhs.uk/]. The codes used to identify kidneys transplants in all-England Hospital Episode Statistics M012, M013, M014, M015, M018 and M019 (OPCS-4).
