# Supplementary material for: Declining comorbidity-adjusted mortality rates in English patients receiving maintenance renal replacement therapy
Source: Kidney Int. 2018 May;93(5):1165–74. doi: 10.1016/j.kint.2017.11.020 (PMC5912929; doi:10.1016/j.kint.2017.11.020)
Supplement: Table S2 — Baseline characteristics of all-England adults being treated for end-stage renal disease, recorded in all-England Hospital Episode Statistics and the UK Renal Registry by year. [file mmc3.pdf]

Supplemental table 2: Baseline characteristics of all-England adults being treated for end-stage renal disease, recorded in all-England Hospital Episode Statistics and the UK Renal Registry by year

	Year Groups							
	2007		2008		2009		2010	
	**HES**	**UK-RR**	**HES**	**UK-RR**	**HES**	**UK-RR**	**HES**	**UK-RR**
**Number of incident RRT patients***	5,412	5,483	5,420	5,626	5,572	5,690	5,501	5,541
**Demographics**								
Female	38%	38%	39%	39%	39%	38%	38%	37%
Median age† (years)	63	64	63	64	63	64	64	64
Ethnicity‡								
Proportion with ethnicity reported	*94%*	*75%*	*94%*	*74%*	*95%*	*78%*	*95%*	*94%*
White	76%	78%	76%	78%	76%	80%	77%	78%
Black	7%	8%	7%	7%	7%	8%	6%	7%
Asian	8%	11%	9%	11%	9%	10%	8%	12%
Others	4%	3%	4%	3%	4%	3%	4%	2%
**Renal characteristics**								
Initial renal replacement therapy modality								
Dialysis	92%	95%	91%	94%	91%	94%	90%	93%
Transplant	8%	5%	9%	6%	9%	6%	10%	7%
Primary renal diagnosis								
Diabetic kidney disease	20%	20%	20%	21%	19%	22%	17%	21%
Glomerulonephritis	14%	10%	15%	11%	17%	11%	23%	11%
Polycystic kidney disease	9%	6%	9%	7%	8%	6%	8%	6%
Other known diagnosis/unknown/unavailable	57%	64%	56%	61%	56%	61%	52%	62%

Date are n or % or median. HES = Hospital Episode Statistics (all-England). UK-RR = UK Renal Registry. Only years with >99% of renal units providing data to UK-RR presented [https://www.renalreg.org/publications-reports/]. *Most recently reported data are presented. †UK-RR 2007 data were derived from England and Wales only. UK-RR 2008-2010 data were from England only. ‡Ethnicity derived from Hospital Episode Statistics used more categories.
