# Supplementary material for: Declining comorbidity-adjusted mortality rates in English patients receiving maintenance renal replacement therapy
Source: Kidney Int. 2018 May;93(5):1165–74. doi: 10.1016/j.kint.2017.11.020 (PMC5912929; doi:10.1016/j.kint.2017.11.020)
Supplement: Table S3 — Baseline characteristics of general population hospital controls, by year. [file mmc4.pdf]

Supplemental table 3: Baseline characteristics of general population hospital controls, by year

	Year Groups							
	Oxfordshire					All-England		
	Oxford Record Linkage Study		Hospital Episode Statistics (Oxfordshire)			Hospital Episode Statistics (All-England)		
	**1970-1990**	**1991-1996**	**2000-2002**	**2003-2005**	**2006-2008**	**2000-2002**	**2003-2005**	**2006-2008**
N	406,897	125,122	86,476	87,000	79,593	1,667,316	1,596,382	1,564,995
**Demographics**								
Female	56.9%	51.8%	51.0%	49.7%	49.6%	50.3%	49.5%	49.2%
Median age (years)	40 (29-57)	41 (30-63)	47 (33-65)	47 (33-66)	47 (33-64)	49 (34-68)	51 (35-69)	51 (35-68)
18 - 40	49.5%	47.3%	39.3%	37.3%	37.2%	36.3%	33.2%	33.3%
40 - 50	15.4%	13.8%	14.5%	15.4%	16.0%	14.0%	14.6%	15.3%
50 - 60	12.4%	10.3%	13.9%	14.3%	14.7%	13.4%	13.8%	13.9%
60 - 70	10.4%	10.4%	12.1%	12.8%	13.2%	12.7%	13.3%	14.0%
70 - 80	8.5%	10.4%	11.7%	11.6%	10.6%	13.6%	14.0%	13.1%
≥80	4.0%	7.8%	8.4%	8.5%	8.4%	9.9%	11.0%	10.3%
**Comorbidities**								
Diabetes	0.8%	1.2%	2.2%	3.1%	4.1%	3.2%	4.5%	5.5%
Vascular	2.0%	2.6%	2.9%	3.3%	3.6%	3.9%	4.3%	4.4%
Non-vascular	3.6%	3.6%	5.7%	6.6%	9.8%	8.2%	9.7%	11.7%

Excludes patients dying within 90 days. Data are n or % or median (IQR). The ‘controls’ were individuals who had been admitted to hospital for any one of a wide range of minor medical or surgical conditions. These included admissions with diagnoses of squint, cataracts, otitis externa/media, varicose veins, hemorrhoids, upper respiratory tract infections, nasal polyps, teeth disorders, inguinal hernia, nail diseases, sebaceous cyst, soft tissue knee complaints, bunions, contraceptive advice, limb fractures, dislocations sprains and strains, minor head injury, superficial injuries or contusions and gallbladder disease, and operations included appendiectomy, dilation and curettage, primary lower limb arthoplasties, tonsillectomy and adenoidectomy. For individuals with more than one control condition, the episode of care which was included in the analysis as the control event was selected at random and any patients entering the renal replacement therapy cohort were excluded.
