# Supplementary material for: Declining comorbidity-adjusted mortality rates in English patients receiving maintenance renal replacement therapy
Source: Kidney Int. 2018 May;93(5):1165–74. doi: 10.1016/j.kint.2017.11.020 (PMC5912929; doi:10.1016/j.kint.2017.11.020)
Supplement: Table S4 — Coding of death categories by International Classification of Disease (ICD) revision. [file mmc5.pdf]

Supplemental table 4: Coding of death categories by International Classification of Disease (ICD) revision

	ICD-10	ICD-9	ICD-8	ICD-7
**Vascular**				
**Cardiac**[†]	I00:I09, I11, I20:I25, I27:I52, R96, R98	390:399, 402, 410:414, 416:429, 7981, 798.2, 798.9	390:399, 402, 410:414, 420:429, 795.2, 796.2	400:416, 420:422, 430:434, 795.2, 795.3
**Non-cardiac vascular**				
Other vascular terms	I10, I14:I15, I26, I70:I84, I86:I99, E10.5, E11.5, E12.5, E13.5, E14.5	401, 405, 415, 440:459, 250.6	400:401, 440:448, 450:458	440:441, 443:445, 450:468
Cerebrovascular	I60:I69	430:438	430:438	330:334
**Non-Vascular**				
**Renal** (non-neoplastic)				
Renal failure[‡]	A98.5, E10.2, E11.2, E12.2, E13.2, E14.2, I12:I13, N17:N19, N25:N27, O08.4, O90.4, P96.0, R39.2, Y60.2, Y61.2, Y622, Y841, T82.4, T82.7, T82.8, T82.9	403:404, 584:589, V45.1, V56.0, V56.8, 250.3, 639.3, 794.4	403:404, 580, 582:584, 792	442, 446, 590, 592:594, 792,
Renal disease[#]	E85, N00:N08, N14:N16[¶], N20:N23, N28:N29, Q60:Q63, M30:M36, C88.0:C88.3, D47.2, D89.0:D89.2, M10.3, O10.2, O10.3, Q27.1, Q27.2,	273, 580:583, 591:593, 710, 753, 2766, 277.3, 652.1, 652.2, 747.6,	446, 581, 591:593, 753, 519.1	522, 591, 601:604, 757
**Cancer**				
Myeloma	C90	203	203	203
Other haematological	C81:C89, C92:C96	200:202, 204:208	200:202, 204:208	200:202, 204:208
Other	C00:C80, C97	140:199, 230:239	140:199, 230:239	140:189, 191:199, 230:239
**Infection**				
Respiratory	J09:J18, J85:J86	480:487, 510, 513	470:474, 480:486, 510, 513	480:493, 518, 521
Genitourinary	N10:N13, N30, N34, N41, N45, N39.0	590, 595, 597, 601, 604, 599.0	590, 595, 597, 601, 604, 680:686, 710, 599.0	600, 605, 607, 611, 614
Other[*]	A00:B99, L00:L08, M00:M03, M46, M49, M73, M86, K35:K37, K57, K61, K65, K80:K81, K83, G00:G09, N70:N77	001:139, 680:686, 711, 730, 540:542, 562, 566:567, 572, 574, 320:326, 614:616	000:136, 720, 540:543, 562, 566, 567, 574, 575, 320:324, 612, 620, 622	001:138, 690:698, 720,730, 550:552, 575, 576, 584, 585, 340:344, 622, 630
**Other/unspecified**	All other ICD-10 codes	All other ICD-9 codes	All other ICD-8 codes	All other ICD-7 codes

[†]Includes codes for sudden death
[‡]Includes codes for AKI, CKD, renal sclerosis, renal failure unspecified, uraemia, diabetes mellitus with renal complications, hypertension with mention of renal failure, misadventures/complication during dialysis. ICD-7 & ICD-8 terms chronic nephritis are also included
[#]Includes terms for glomerular disease, tubulo-interstitial disorders, obstructive uropathies, urolithiasis, congenital abnormalities affecting the kidney, amyloid, paraproteinaemias, fluid overload and autoimmune/vasculitis/connective tissue disorders
[¶]Excludes urinary tract infection ("N160") which is included in the infection category
[*]Includes skin and soft tissue, bone and joint, abdominal, central nervous system, gynaecological (not during puerperium) infection and generic infection chapters.
