# Supplementary material for: Declining comorbidity-adjusted mortality rates in English patients receiving maintenance renal replacement therapy
Source: Kidney Int. 2018 May;93(5):1165–74. doi: 10.1016/j.kint.2017.11.020 (PMC5912929; doi:10.1016/j.kint.2017.11.020)
Supplement: Table S5 — Coding of comorbidity by International Classification of Disease (ICD) diagnoses and Office of Population Censuses and Surveys (OPCS) procedures. [file mmc6.pdf]

Supplemental table 5: Coding of comorbidity by A) International Classification of Disease (ICD) diagnoses and Office of Population Censuses and Surveys (OPCS) procedures

A) Diagnostic codes

	ICD-10	ICD-9	ICD-8	ICD-7
**Diabetes**	E10:E14	250	250	260
**Vascular**				
Major coronary disease	I21:I23, I252, I25.6	410, 412, 414.8	410, 412	420
Congestive heart failure	I42:I43, I50, I11.0, I13.0, I13.2, I25.5, P29.0	402, 404, 425, 428	402, 404, 425, 427	434
Cerebrovascular disease	I60:69, G45, G46, H34.0	430:431, 434:436, 438, 432.0, 432.1	430:431, 433:436	330:334
Peripheral vascular disease	I70:I74, K55.0, K55.1, K55.8, K55.9, R02, Z95.8, Z95.9, E10.5, E11.5, E12.5, E13.5, E14.5	440:444, 250.6	440:445	450:454
**Non-Vascular**				
Liver	B18, K70:K76, I85, I86.4, I98.2	570:573	571, 573	581
Cancer	C00:C97	140:208, V10	140:239	140:239
COPD*	J41:J47, J60:J67, J68.4, J70.1,J70.3, J84, I27.8, I27.9	416.8, 416.9, 491:496, 500:506, 515, 508.1	490:493, 515:518	241, 501:502, 523:526, 527.1
Peptic ulcer disease	K25:K28	531:534, 530.3	531:534	540:542
Hemi- or paraplegia	G81:G83, G04.1, G11.4, G80.1, G80.2	342:344	343:344	351:352
Connective tissue disease	M05: M06, M30:M36	517, 710, 714, 446.0, 446.4, 446.7, 447.6	712, 716, 734, 695.4, 696.0	722

B) Procedural codes

	OPCS-4	OPCS-3	OPCS-2	OPCS-1
**Vascular**				
Major coronary disease[‡]	K40:K47, K49, K50, K75	304.1, 304.3	304.1, 304.2	312
Peripheral vascular disease[¶]	X07:X12, J10.4, K33, L04, L12:L13, L16, L18:L21, L25:L31, L33:L35, L37:L39, L41:L54, L56:L60, L62:L63, L65:L66, L68:L71, L76, L89, L97, O20	304, 320:321, 325, 552, 822:826, 861:866, 870:871, 873:875, 878, 880:884, 887:888, 890	860:875, 884, 8811	880:886

[*] COPD=chronic obstructive pulmonary disease
[‡] Includes codes for coronary artery bypass surgery or percutaneous coronary interventions (angioplasty +/- stent)
[¶] Includes codes for (non-traumatic) limb amputations and non-coronary arterial interventions
