# Supplementary material for: Declining comorbidity-adjusted mortality rates in English patients receiving maintenance renal replacement therapy
Source: Kidney Int. 2018 May;93(5):1165–74. doi: 10.1016/j.kint.2017.11.020 (PMC5912929; doi:10.1016/j.kint.2017.11.020)
Supplement: Table S6 — Baseline characteristics of end-stage renal disease populations used for standardization. [file mmc7.pdf]

Supplemental table 6: Baseline characteristics of end-stage renal disease populations used for standardization

	1970 - 2008 'average' RRT population	2003 - 2008 RRT population
N	3,242	29,552
**Demographics**		
Female	39.1%	38.2%
Median age (years)	55 (42-68)	62 (48-73)
18 - 40	22.0%	13.8%
40 - 50	16.9%	13.2%
50 - 60	19.9%	16.9%
60 - 70	20.4%	22.8%
70 - 80	17.0%	24.3%
≥80	3.9%	9.0%
**Comorbidities**		
Diabetes	17.8%	32.3%
Vascular	17.8%	27.5%
Non-vascular	15.7%	26.3%

Excludes patients dying within 90 days. Data are n or % or median (IQR). RRT = Renal replacement therapy.
