# Supplementary material for: Declining comorbidity-adjusted mortality rates in English patients receiving maintenance renal replacement therapy
Source: Kidney Int. 2018 May;93(5):1165–74. doi: 10.1016/j.kint.2017.11.020 (PMC5912929; doi:10.1016/j.kint.2017.11.020)
Supplement: Table S7 — Diagnostic and procedural codes used to identify general population controls. [file mmc8.pdf]

Supplemental Table 7: Diagnostic and procedural codes used to identify general population controls

Diagnoses	ICD-10	ICD-9	ICD-8	ICD-7
**Ophthalmic**				
Squint	H49:H51	378	373	384
Cataract	H25	366	374	385
**General surgical**				
Gallbladder disease	K80:K81	574:575	574:575	584:585
Hernia	K40	550	550	560:561
Varicose veins	I84	455	455	461
Haemorrhoids	I83	454	454	460
**Otorhinolaryngology**				
Otitis externa/media	H60:H67	380:382	380:382	390:392
Nasal polyp/deflecting septum	J33, J34.2	470:471	504:505	514:515
**Injuries**				
Limb fractures	S42, S52, S62, S82, S92	810:816, 823:826	810:816, 823:826,	810:816, 823:826,
Dislocations sprains and strains	S03, S13, S23, S33, S43, S53, S63, S73, S83, S93	830:839, 840:848	830:839, 840:848,	830:839, 840: 848,
Head injury	S06	850:854	850:854,	852:856
Superficial injury and contusion	S00, S10, S20, S30, S40, S50, S60, S70, S80, S90	910:919, 920:924	910:918, 920:929	910:918, 920:929
**Miscellaneous**				
Nail diseases	L60	703	703	712
Sebaceous cyst	L72.1	7062	7062	7142
Knee-internal derangement	M23	717	724	734
Bunion	M20.1	7271	730	740
Contraception management	Z30	V25	Y43	-
Upper respiratory tract infection	J00:J06	460:466	460:466	470:475
Teeth disorders	K00:K03	520:521	520:521	530:535

Procedures/Operations	OPCS-4	OPCS-3	OPCS-2	OPCS-1
Appendiectomy	H01:H03	441:444	441:444	441
Dilation and curettage	Q10.3:Q11.4	703:704	703:704	731:732
Total hip replacement	Y37:Y39	810	810	-
Total knee replacement	Y40:Y42	812	812	-
Tonsillectomy/adenoidectomy	E20, F34, F36	230:236	230:236	260:264

ICD=International Statistical Classification of Diseases and Related Health Problems. OPCS=Office of Population Censuses and Surveys Classification of Surgical Operations and Procedures.
