# Supplementary material for: Declining comorbidity-adjusted mortality rates in English patients receiving maintenance renal replacement therapy
Source: Kidney Int. 2018 May;93(5):1165–74. doi: 10.1016/j.kint.2017.11.020 (PMC5912929; doi:10.1016/j.kint.2017.11.020)
Supplement: Figure S1 — Age- and sex-standardized 3-year mortality rates in newly treated end-stage renal disease patients and general population hospital controls in Oxfordshire. [file mmc9.pdf]

Supplemental figure 1: Age and sex standardized three−year mortality rates in new treated end−stage renal disease patients and general population hospital controls in Oxfordshire

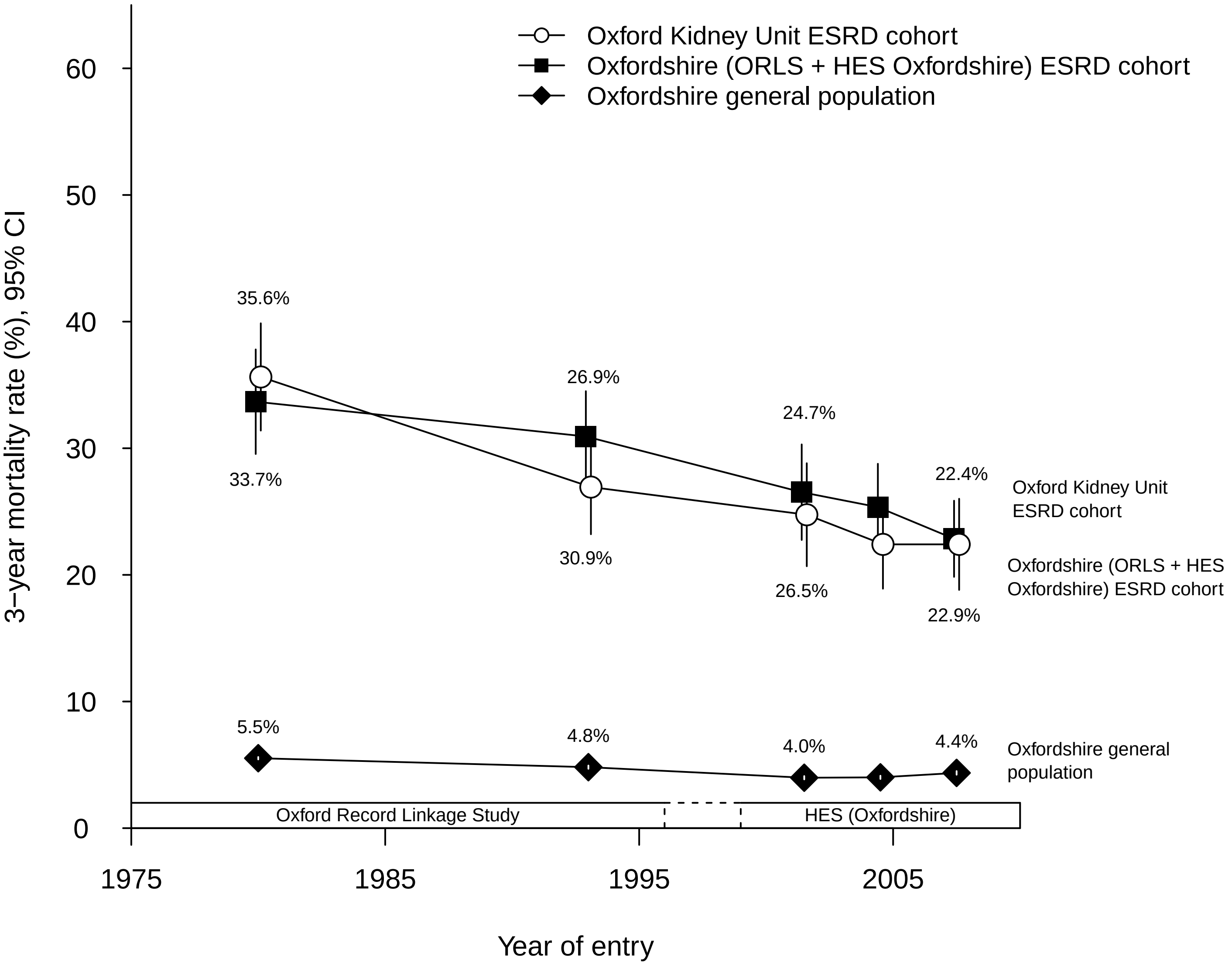

Standardized to the age and sex structure of an 'average' 1970−2008 renal replacement therapy population (see Supplemental Table 6 for characteristics). Excludes patients dying within 90 days. ESRD = End−stage renal disease. HES = Hospital Episode Statistics (Oxfordshire). OKU = Oxford Kidney Unit. ORLS = Oxford Record Linkage Study. Year of entry is year of starting renal replacement therapy or year of relevant general population hospital controls' admission. Hospital Episode Statistics restricted to patients with residency within the Oxford Record Linkage region of Oxfordshire, Berkshire, Buckinghamshire and Northamptonshire. Rates plotted at midpoint of each year group. Estimated three−year mortality rates (%) for OKU are quoted above circles, while ORLS rates are quoted below squares.
