# Supplementary material for: Declining comorbidity-adjusted mortality rates in English patients receiving maintenance renal replacement therapy
Source: Kidney Int. 2018 May;93(5):1165–74. doi: 10.1016/j.kint.2017.11.020 (PMC5912929; doi:10.1016/j.kint.2017.11.020)
Supplement: Figure S2 — Baseline characteristics of newly treated end-stage renal disease patients, by year. [file mmc10.pdf]

**Supplemental figure 2: Baseline characteristics of new treated end−stage renal disease patients, by year**

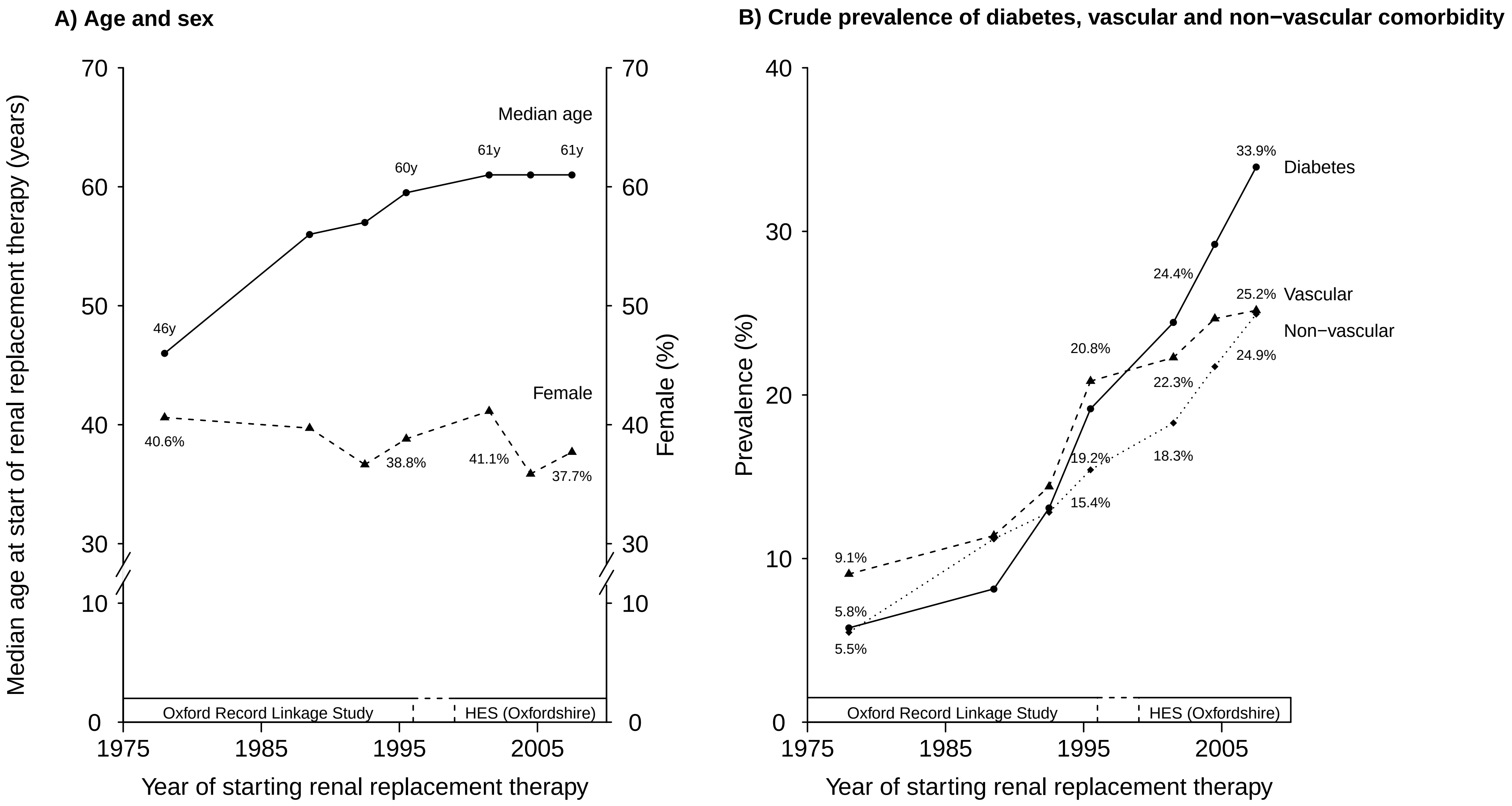


Excludes patients dying within 90 days. HES = Hospital Episode Statistics (Oxfordshire). Results are plotted at midpoint for each year group. For this figure, the Oxford Record Linkage Study includes 4 year groups (1970−85, 1986−90, 1991−93, 1994−96).
