# Supplementary material for: Declining comorbidity-adjusted mortality rates in English patients receiving maintenance renal replacement therapy
Source: Kidney Int. 2018 May;93(5):1165–74. doi: 10.1016/j.kint.2017.11.020 (PMC5912929; doi:10.1016/j.kint.2017.11.020)
Supplement: Figure S3 — Crude and standardized 3-year mortality rates in newly treated end-stage renal disease patients and general population hospital controls, by year and reference population. [file mmc11.pdf]

Supplemental figure 3: Crude and standardized three−year mortality rates in new treated end−stage renal disease patients and general population hospital controls, by year and reference population

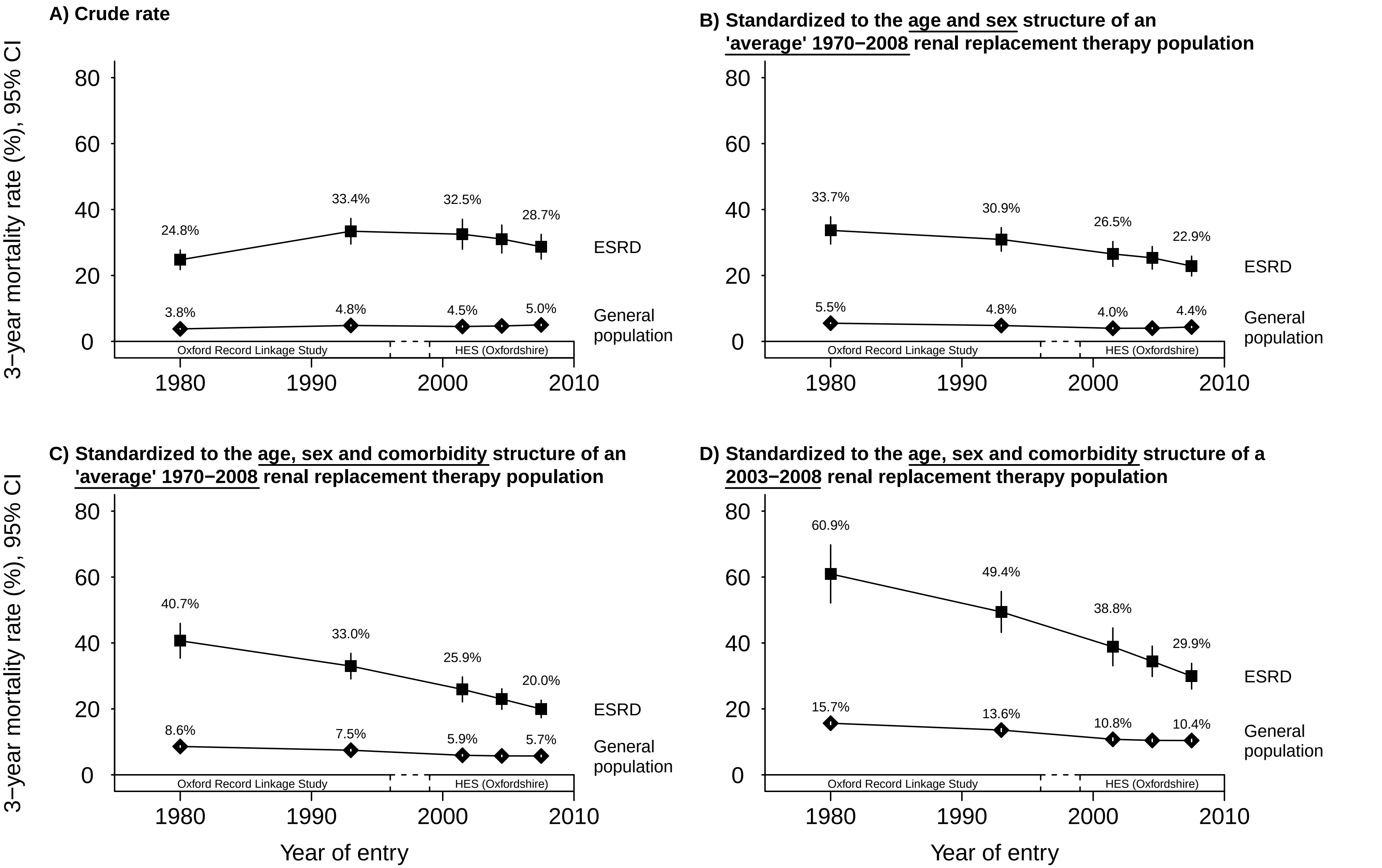

See Supplemental Table 6 for characteristics of reference populations. Excludes patients dying within 90 days. ESRD = End−stage renal disease. HES = Hospital Episode Statistics (Oxfordshire). ORLS = Oxford Record Linkage Study. Year of entry is year of starting renal replacement therapy or year of relevant general population hospital controls admission. Rates plotted at midpoint of each year group.
