# Supplementary material for: Declining comorbidity-adjusted mortality rates in English patients receiving maintenance renal replacement therapy
Source: Kidney Int. 2018 May;93(5):1165–74. doi: 10.1016/j.kint.2017.11.020 (PMC5912929; doi:10.1016/j.kint.2017.11.020)
Supplement: Figure S4 — Standardized 1- to 5-year survival probabilities in newly treated end-stage renal disease patients. [file mmc12.pdf]

Supplemental figure 4: Standardized one− to five−year survival probabilities in new treated end−stage renal disease patients

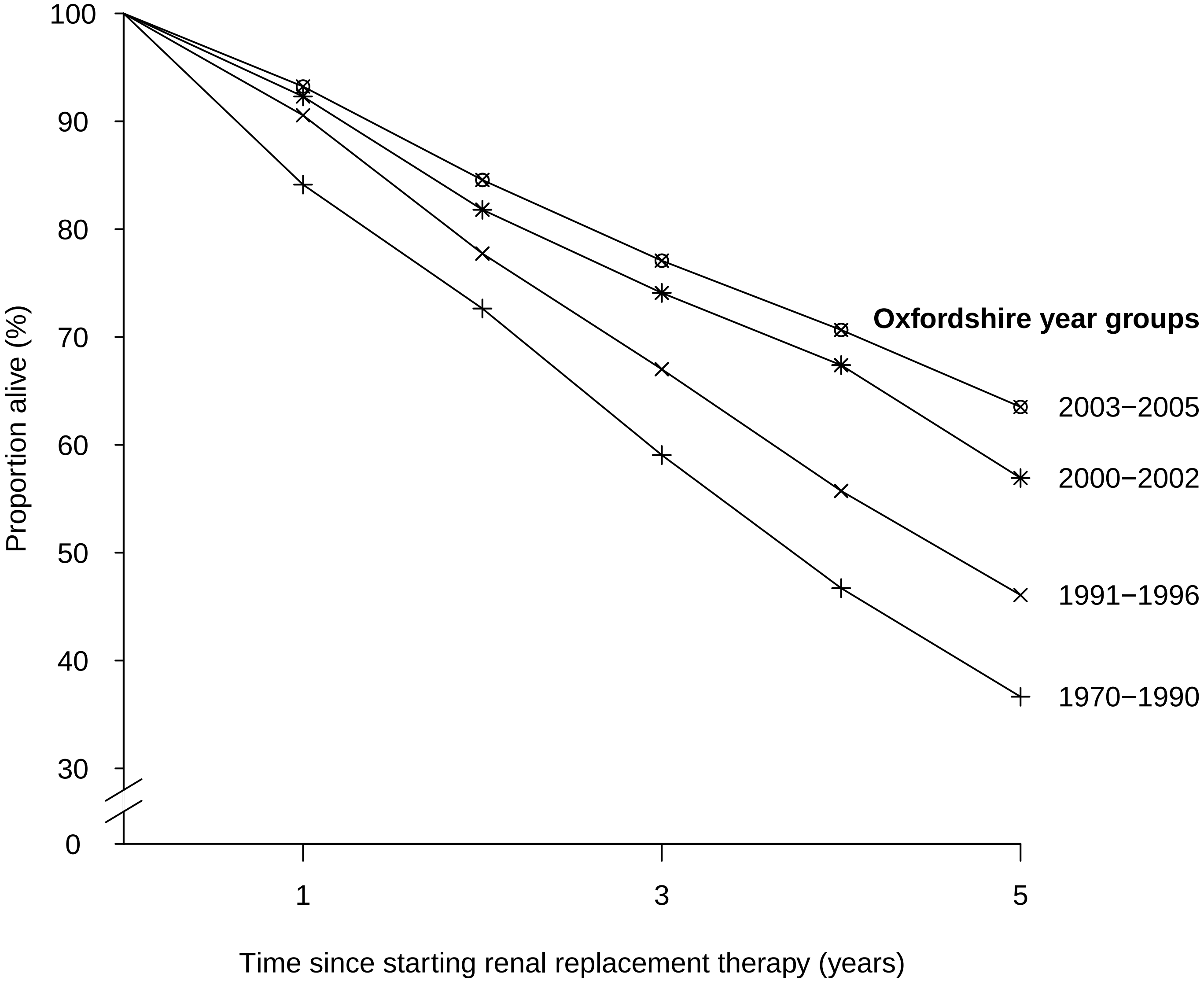

**Oxfordshire year groups**			
**2003−2005 †**	93.2% (91.6%−94.9%)	77.1% (73.9%−80.2%)	63.5% (59.4%−67.7%)
**2000−2002 †**	92.3% (90.4%−94.2%)	74.1% (70.3%−77.9%)	56.9% (51.9%−61.9%)
**1991−1996 ***	90.6% (88.6%−92.5%)	67.0% (63.1%−70.9%)	46.1% (40.9%−51.2%)
**1970−1990 ***	84.1% (80.9%−87.4%)	59.1% (53.6%−64.5%)	36.6% (29.8%−43.5%)

Survival probabilities are 100 minus the adjusted mortality rates, standardized by age, sex and comorbidities to an 'average' 1970−2008 renal replacement therapy population (see Supplemental Table 6 for characteristics). Excludes patients dying within 90 days. 95% confidence intervals included in parentheses. *Oxford Record Linkage Study. † Hospital Episode Statistics (Oxfordshire).
