# Supplementary material for: Declining comorbidity-adjusted mortality rates in English patients receiving maintenance renal replacement therapy
Source: Kidney Int. 2018 May;93(5):1165–74. doi: 10.1016/j.kint.2017.11.020 (PMC5912929; doi:10.1016/j.kint.2017.11.020)
Supplement: Figure S5 — Treated end-stage renal disease cohort derivation (all-England HES 2000–2008). [file mmc13.pdf]

Supplemental figure 5: Treated end-stage renal disease cohort derivation (all-England HES 2000-2008)

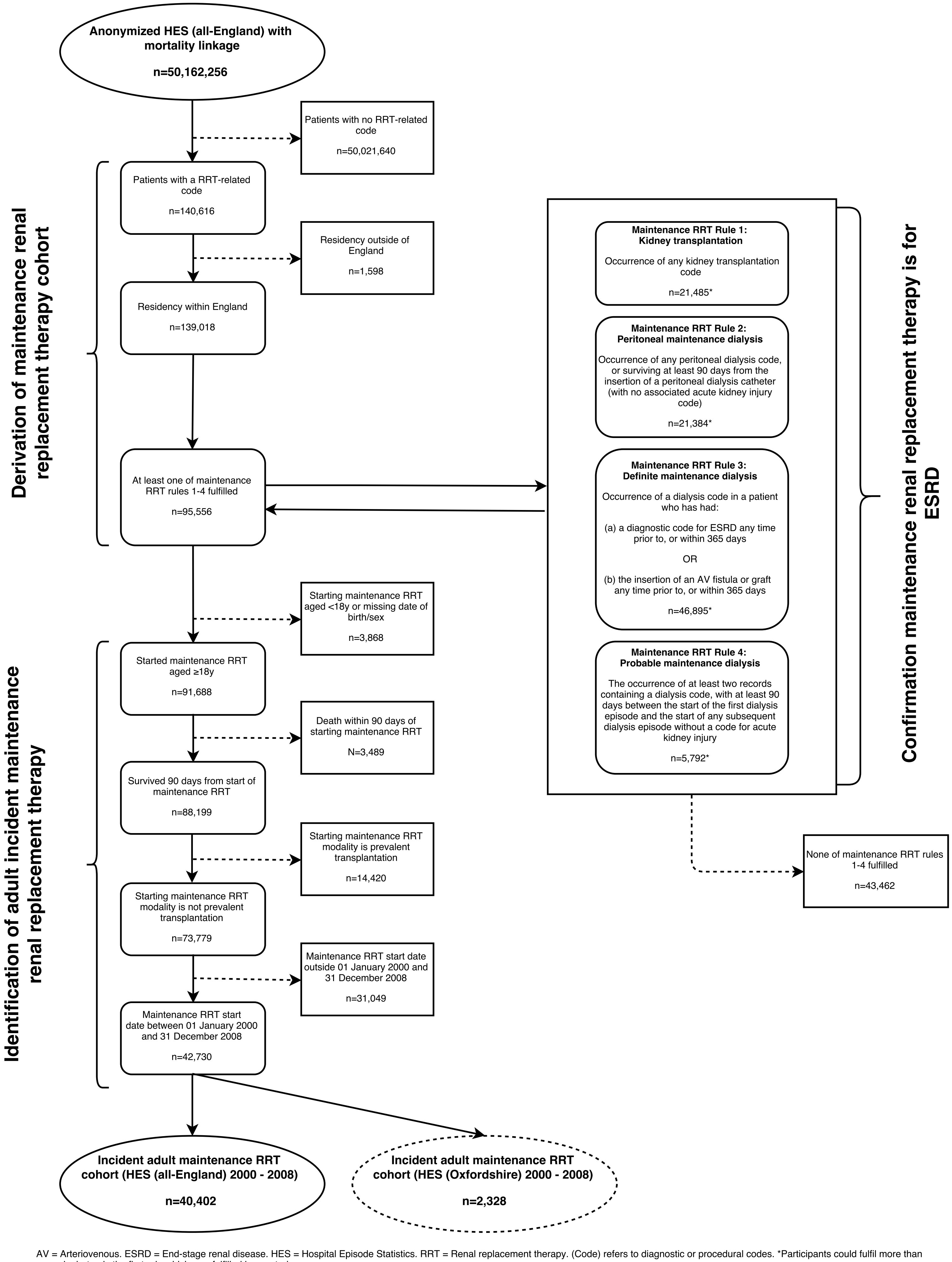

AV = Arteriovenous. ESRD = End-stage renal disease. HES = Hospital Episode Statistics. RRT = Renal replacement therapy. (Code) refers to diagnostic or procedural codes. *Participants could fulfil more than one rule, but only the first rule which was fulfilled is counted.
