# Supplementary material for: Declining comorbidity-adjusted mortality rates in English patients receiving maintenance renal replacement therapy
Source: Kidney Int. 2018 May;93(5):1165–74. doi: 10.1016/j.kint.2017.11.020 (PMC5912929; doi:10.1016/j.kint.2017.11.020)
Supplement: Figure S6 — Treated end-stage renal disease cohort derivation (Oxford Record Linkage Study 1970–1996). [file mmc14.pdf]

Supplemental figure 6: Treated end-stage renal disease cohort derivation (Oxford Record Linkage Study 1970-1996)

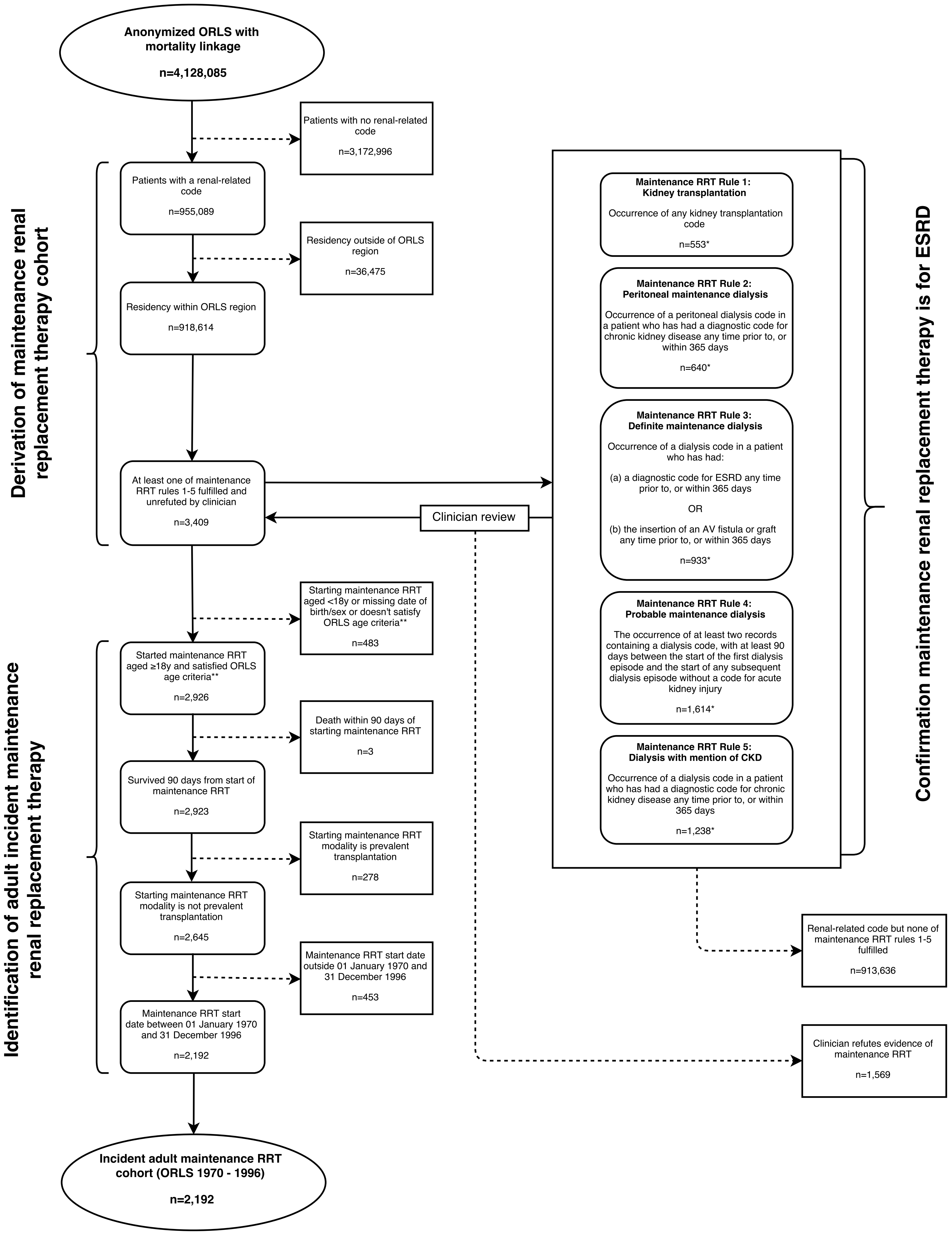

AV = Arteriovenous. CKD = Chronic kidney disease. ESRD = End-stage renal disease. ORLS = Oxford Record Linkage Study. RRT = Renal replacement therapy. (Code) refers to diagnostic or procedural codes. *Participants could fulfil more than one rule, but only the first rule which was fulfilled is counted. **If RRT start date between 1970 - 1974 then age<60, if RRT start date between 1975 - 1979 then age<70, and if RRT start date between 1980 - 1984 then age<80. To ensure reliable derivation of the ORLS cohort, a broad range of diagnostic and procedural codes was used and combined with clinician review of individual patient records.
