# Supplementary material for: Declining comorbidity-adjusted mortality rates in English patients receiving maintenance renal replacement therapy
Source: Kidney Int. 2018 May;93(5):1165–74. doi: 10.1016/j.kint.2017.11.020 (PMC5912929; doi:10.1016/j.kint.2017.11.020)
Supplement: Figure S7 — Standardized 3-year mortality rates in newly treated end-stage renal disease patients and general population hospital controls, by year and matching method. [file mmc15.pdf]

Supplemental figure 7: Standardized three−year mortality rates in new treated end−stage renal disease patients and general population hospital controls, by year and matching method

Oxfordshire

	% change since 1980 (95% CI)		% change since 2000 (95% CI)	
**Matched ESRD cohort***	**−52%**	(−62%,−43%)	**−24%**	(−39%,−8%)
**All ESRD cohort**	**−51%**	(−60%,−41%)	**−23%**	(−38%,−8%)
**Matched hospital controls***	**−43%**	(−65%,−21%)	**−7%**	(−39%,25%)
**All hospital controls**	**−34%**	(−36%,−31%)	**−3%**	(−8%,1%)

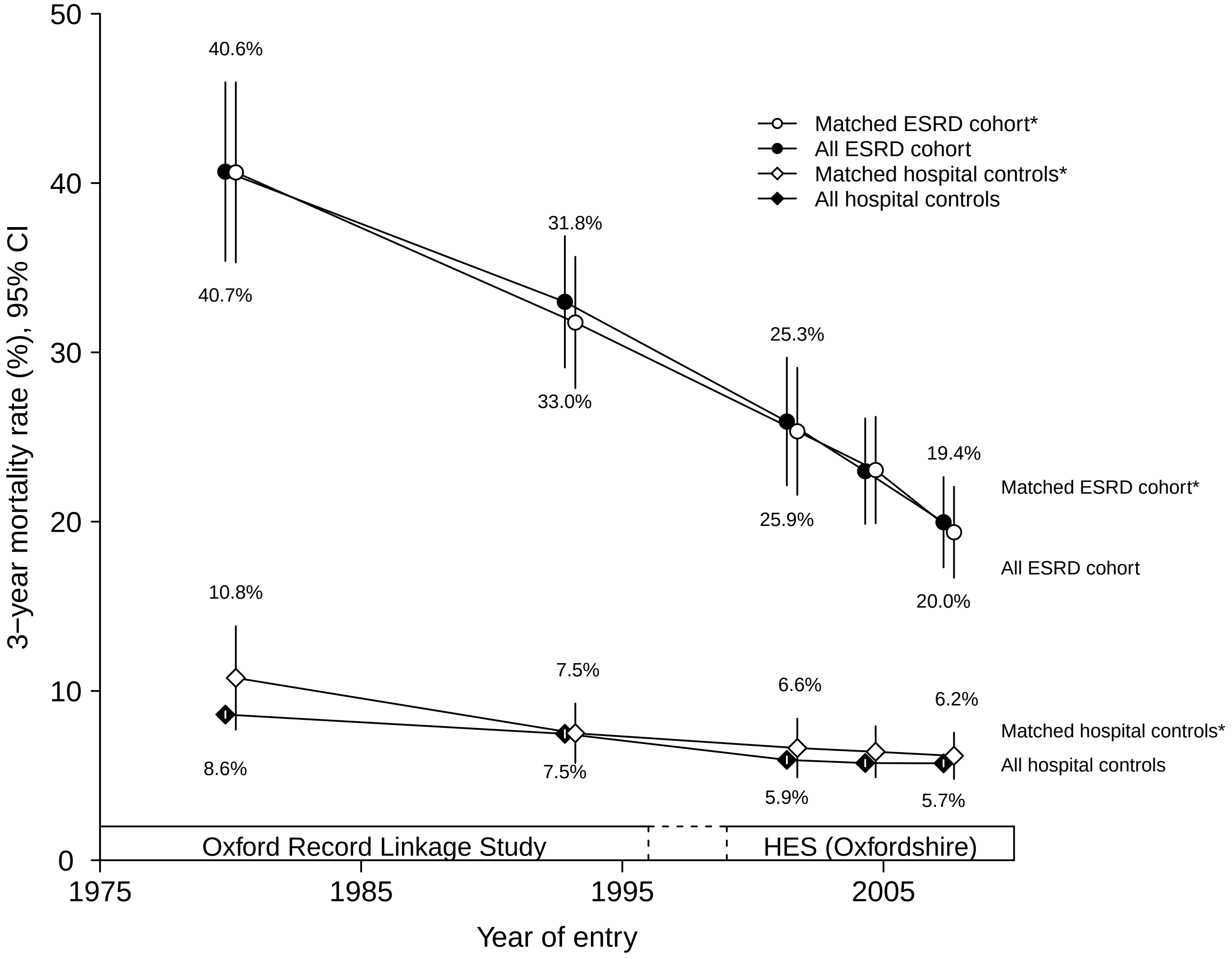

Standardized to the age, sex and comorbidity structure of an 'average' 1970−2008 renal replacement therapy population (Supplemental Table 6 for characteristics). Excludes patients dying within 90 days. ESRD = End−stage renal disease. HES = Hospital Episode Statistics. Year of entry is year of starting renal replacement therapy or year of relevant general population hospital controls' admission. Rates plotted at midpoint of each year group. *Matched by age, sex and comorbidity. 125 ESRD patients for whom a matching hospital control could not be identified were excluded from the analyses.
